# Beyond integrated care: challenges on the way towards population health management

**DOI:** 10.5334/ijic.2424

**Published:** 2015-12-15

**Authors:** Jeroen N. Struijs, Hanneke W. Drewes, K. Viktoria Stein

**Affiliations:** Center for Nutrition, Prevention and Health Services, Department of Quality of Care and Health Economics, National Institute of Public Health and the Environment (RIVM), Bilthoven, The Netherlands; National Institute of Public Health and the Environment (RIVM), Bilthoven, The Netherlands; Head of Education and Training, International Foundation for Integrated Care

Integrated care has long emerged as a viable approach to overcome deficiencies in the care management for people with complex health care needs such as chronic diseases, disabling conditions, serious mental illness, and medically fragile children and the frail elderly, while at the same time improving efficiency, quality and effectiveness of the health services provided. The focus thus has been on better coordination and integration among health care sectors to manage specific (chronic) diseases. However, it has become evident that in order to provide truly population-centred services that improve population health, the scope of integrated care needs to be expanded to bridge the gaps not only within the health system, but also between the health and social systems, among others. Consequently, initiatives are emerging internationally that have a broader focus than integrating health care. These initiatives aim to integrate services across health care, prevention, social care and welfare, and are often labelled as population (health) management [1]. For instance, in the Netherlands, several regional partnerships originated in which care providers, insurers and stakeholders like municipalities and representatives of citizens participate [2]. Similar trends can be observed in other countries, like Germany (e.g. Gesundes Kinzigthal [3]), England (e.g. Torbay [4]) and the USA (e.g. Accountable *Community* Organization [5]), where initiatives have developed, which all take a population health management focus. The scope of these initiatives reflects a wide array of integrated care concepts. All concepts need to overcome common barriers and challenges in order to be successful.

First, successful governance arrangements need to be created. In all initiatives, multiple actors are involved, with their own organisational interests, leading to varying governance arrangements. How to best arrange these new governance arrangements, which need to include elements of accountability, oversight and distributed leadership, while at the same time considering the national, regional and local context, is still widely discussed and yet to be resolved. For sustainable change, this question needs to move into the focus of decision makers, as good governance arrangements are a prerequisite for successful implementation of population health management [6].

Second, within population health management initiatives questions arise about how to engage the population they serve. Various strategies to actively involve the local community have already been launched, such as online ‘communities’, patient representatives as board members of health services, and even new entities led by citizens, which serve as integrator. These tools and the definitions of the concepts behind them vary considerably in scale and scope. A distinction needs to be made between individual patient engagement versus broader community involvement [7]. Evidence about the effectiveness of various patient and population engagement strategies has confirmed their usefulness for improving health outcomes and quality of life of patients, families and communities [7,8]. However, it has also become clear that there needs to be a combination of strategies applied in order to truly support population health management. In addition, more insight is needed to ascertain what works for whom in which context to successfully involve the community.

Third, there is an ongoing debate about the appropriate payment models for these initiatives. Alternatives, like global budgets including a two-sided shared savings model, which are tied to quality improvements, analogous to the Alternative Quality Contract [9,10], receive scrutiny in this debate. An even more disruptive payment model has emerged in England, where budgets of social care and NHS budgets of Clinical Commissioning Groups are integrated within the ‘Manchester Devolution’ initiative [11]. Similar efforts have been implemented in Scotland and Canterbury (New Zealand), where the local health and social care budgets and authorities have been merged and now need to work on integrating budgets, structures and services. By looking at initiatives experimenting with alternative payment models, lessons can be learned on how to shift financial and clinical accountability from payers towards (groups of) care providers (and potentially in the near future also citizens) in order to incentivise these providers to improve population health, quality of care and reduce costs growth.

Fourth, methodological issues remain on how to evaluate thesecomplex interventions. Current methodologies are insufficient indealing with the complexity of these initiatives, and their interaction with national, regional and local contextual factors. As outlined by Evers and Paulus [12] and Struijs [13] in previous editorials, improvement and refinement of innovative mixed-methods research, which can disentangle these interactions, are needed before ‘transferable lessons’ for other regions or countries can be derived. Experiences and knowledge of these applied methodologies, as, for example, ‘realist evaluations’, are of interest. Additionally, we need to find out the right measures for quantifying the interrelated aims (i.e. the domains population health, quality and costs). More innovative population-centred measures, such as positive health [14] and societal participation and low value care measures are of relevance, though scarcely measured at population level. More efforts are needed to validate these innovative measures on a population level. Ideally, these population-centred measures can also be tied to care provider’s payment models which might foster the transparency in the quality of services delivered.

To address the abovementioned challenges and remaining issues, International Foundation for Integrated Care (IFIC) is setting up a Special Interest Group (SIG) Population Health Management. The kick-off meeting for this SIG will be at the ICIC16 in Barcelona, where the most prominent issues will be discussed and an inventory regarding priority challenges will be compounded. This will result in a ‘research agenda’ for the coming years, during which the SIG will act as a platform in order to connect interested researchers, policy makers, payers and communities in the field of population health management. The research challenges will serve as a basis for projects, position papers and other relevant research activities. In addition, upcoming evidence and case studies will be shared among the SIG members. By doing so, IFIC continues to serve as a central and authoritative voice in the field of innovative initiatives integrating prevention, health care and social care.

If you are interested to join the SIG Population Health Management, please contact Viktoria Stein (email: viktoriastein@integratedcarefoundation.org).
